# A meta-analysis of the correlation between obstructive sleep apnea syndrome and renal injury

**DOI:** 10.1007/s11255-024-04160-z

**Published:** 2024-07-24

**Authors:** Linghong Yang, Bo Liu, Feimin Zhao, Zhangning Zhou

**Affiliations:** 1 Department of General Practice, Huzhou Central Hospital, Affiliated Central Hospital Huzhou University, Huzhou, 313000 ZheJiang China; 2 Department of Nephrology, Huzhou Central Hospital, Affiliated Central Hospital Huzhou University, Huzhou, 313000 ZheJiang China; 3 Department of Geriatrics, Huzhou Central Hospital, Affiliated Central Hospital Huzhou University, Huzhou, 313000 ZheJiang China; 4 Department of Geriatrics, Huzhou Central Hospital, Affiliated Central Hospital Huzhou University, 1558 Sanhuan North Road, Huzhou, 313000 ZheJiang China

**Keywords:** Obstructive sleep apnea syndrome, Renal injury, Correlation, Meta-analysis

## Abstract

**Objective:**

To conduct a meta-analysis on the correlation between obstructive sleep apnea syndrome (OSAS) and renal injury.

**Methods:**

Literature search was carried out in PubMed, Embase and Ovid-Medline databases between the date of database establishment and June 30th 2024. The keywords included obstructive sleep apnea hypopnea syndrome, sleep apnea hypopnea syndrome, renal injury, and correlation. Two researchers 1st independently screened the titles and abstracts based on the eligibility criteria, then extracted the data and evaluated the quality, and used Review Manager 5.3 for data processing. All analysis methods were based on PRISMA.

**Results:**

Finally, 8 studies that matched the inclusion criteria were included, and the relationship between obstructive sleep apnea syndrome and serum cystatin C was analyzed. The homogeneity test showed (*P* < 0.01, *I*^*2*^ = 98%), and from the meta-analysis results, it could be known that the level of serum cystatin C in sufferers with OSAS was obviously greater than the control one (OR = 1.12, 95% CI 0.96–1.28, *P* < 0.01). The relationship between OSAS and serum creatinine was analyzed, and homogeneity test showed (*P* < 0.01, *I*^*2*^ = 96%). From the meta analysis result, it could be known that the serum creatinine level of obstructive sleep apnea syndrome was obviously greater than the control one (OR = 1.01, 95% CI 0.85 ~ 1.17, *P* < 0.01). The relationship between obstructive sleep apnea syndrome with serum urea nitrogen was analyzed, and homogeneity test showed (*P* < 0.01, *I*^*2*^ = 91%). From the meta-analysis results, it could be known that serum urea nitrogen of OSAS was obviously greater than the control one (OR = 1.38, 95% CI 01.17 ~ 1.59, *P* < 0.01).

**Conclusions:**

Eight articles have been included to determine the correlation between obstructive sleep apnea syndrome and renal injury, and it has been found that obstructive sleep apnea syndrome is closely related to renal injury, and the two may be risk factors for each other.

## Introduction

OSAS is apnea and hypoventilation caused by repeated upper airway collapse and obstruction during sleep, resulting in sleep structure disturbance, intermittent hypoxia, hypercapnia, increased intrathoracic pressure fluctuations, autonomic nervous system dysfunction and inflammatory activation [1, 2]. Clinically, it can be manifested as snoring, snoring loud and irregular, choking or holding awake at night, sleep disturbance, drowsiness during the daytime, memory loss, and abnormal behavior. The prevalence of obstructive sleep apnea hypopnea syndrome in China is 3–4%, and it is as high as 20–40% for people over 65 years old. 3000 people in the world die due to obstructive sleep apnea hypopnea syndrome every day [3, 4]. Patients who have been diagnosed with obstructive sleep apnea syndrome, it means that their body will repeatedly suffer from hypoxia while sleeping, and the kidney is a very sensitive organ to hypoxia, so repeated hypoxia during sleep may lead to renal injury. In addition, sufferers with OSAS are usually prone to a series of problems in health, such as diabetes, obesity, and high blood pressure, and the latter is also a common risk factor for renal injury [5–7]. Therefore, the kidneys of sufferers with OSAS who have multiple damage factors are more prone to damage. In the study of renal injury and apnea, the researchers tested 34 sufferers with OSAS, six of whom were found to have significant proteinuria, and three of them had reached the category of nephropathy. In the later follow-up, it was found that proteinuria was also significantly reduced in four of the patients after the treatment of apnea syndrome [8]. In recent years [9], studies have shown that serum cystatin C levels in patients with simple OSAS are higher than those in the normal control group, and urine *N*-acetyl levels are higher *β*-D-glucosamine was elevated compared to the normal control group, and the levels of cystatin C and urine *N*-acetyl-B-D glucosamine were affected by the Apnea Hyponea Index (AHI), which was positively correlated with AHI. This indicates that renal damage in OSAS patients is mainly related to respiratory arrest and hypopnea. Patients with moderate to severe OSAS may experience an increase in serum creatinine, urea nitrogen, and urinary y-glutamyltransferase levels. However, there is no consensus on whether OSAS can cause kidney damage, and some people believe that OSAS does not cause kidney damage. It can be seen that the fluid in the body of patients with chronic kidney disease will be accumulated with the aggravation of the disease, which will aggravate the occurrence of apnea symptoms. Therefore, it can be seen that kidney safety is closely related to sleep quality. Therefore, this study collected the research reports about obstructive sleep apnea syndrome and kidney injury, and used meta-analysis for evaluation of the correlation between the 2 groups of illnesses.

## Data and methods

### Search strategy

The study about obstructive sleep apnea syndrome and renal injury was included. To identify eligible original articles, a series of computerized databases, including PubMed, EMBASE, Web of Science, and CNKI’s Medline were searched using the following key words: “Obstruction Sleep Apnea Syndrome”, “ Kidney Injury”, “Obstructive Sleep Apnea Hypopnea Syndrome”, “Obstructive Sleep Apnea”, “OSAS”. The articles were searched in computerized databases up to June 30th 2024, regardless of language. In addition, a manual search of the references and related reviews included in the study was conducted to locate other relevant articles in the public database that were not retrieved according to the search formula.

### Inclusion and exclusion criteria

Inclusion criteria: (1) the controlled trials in English or Chinese in a random way; (2) patients who were clearly diagnosed with obstructive sleep apnea syndrome; (3) the studies that all members of the research group had excluded any predisposing factors that might be related to the occurrence of OSAS (e.g., Barrett’s esophagus and asthma); (4) studies limited to humans, published in English and others language, containing raw data, and presented in abstract or full text; (5) researches that clearly defined the research one and the control one and their members, and had observational data; (6) there was no missing experimental data, the sample size was accurate, and the data between the research groups were complete.

Exclusion criteria: (1) Review literature and other non-primary literature; (2) literature that there was no literature extraction data; (3) patients complicated with hematological diseases and liver insufficiency; (4) studies that did not clearly define diagnostic criteria for obstructive sleep apnea syndrome and kidney injury; (5) duplicate publications; (6) patients with only a single diagnosis of snoring, sleep disturbance or sleep disturbance were excluded.

### Literature screening and data extraction

Two investigators separately screened titles and abstracts based on eligibility criteria before extracting data and assessing the quality. With the inconsistent assessment results, other researchers were consulted to resolve discrepancies according to the original data. If the title and abstract could meet the given requirements, it could search the full text and perform data extraction. Document management adopted Note express 2.0 and the duplicate documents were deleted. Literature search was carried out in strict conformity with the above inclusion and exclusion criteria, and relevant literature was traced. Two investigators separately extracted relevant information using predefined data extraction tables. The extracted information included author, the year of publication, sample size, age, gender, country, and renal function indicators. If no data can be found in the original articles, the authors could be contacted by e-mail for information. Relevant transformations were performed using the Cochrane Review Handbook for unavailable data.

### Methodological quality evaluation of literature

The “Risk of Bias Assessment” recommended by the Cochrane Handbook of Systematic Reviews (version 5.3) was used for quality evaluation, in order to improve the literature review quality. The evaluation included the following seven items: (1) the stochastic methods; (2) The concealment of allocation; (3) blind method applied between patients and researchers; (4) blind method applied in effect evaluation. For other biases in item 7 above RCTS, “satisfaction” meant smaller bias. “Unsatisfactory” meant that the deviation was large. The selected papers’ quality was assessed based on the Newcastle–Ottawa Scale (NOS) recently recommended in the Practice Guidelines for Systematic Reviews, which was the most reliable tool for assessing the quality of cross-sectional or cohort studies in systematic reviews. The studies with NOS scores were classified into inferior quality (zero to three scores), medium quality (four to six scores) and good quality (more than seven scores).

### Statistical methods

This systematic review used Review Manager 5.3 for data processing, and the test level was 0.05. *I*^2^ < 50% and *P* > 0.05 indicated that there was no statistical heterogeneity among the trials, and the Meta-analysis was carried out using the fixed impact model. However, *I*^2^ ≥ 50% and *P* ≤ 0.05 it could be seen that here was statistical heterogeneity among trials, and random impacts model was chosen for meta-analysis. The comprehensive impact size of the 2 groups of evaluation index data was the OR value and its 95% CI and a forest diagram was drawn to show the research conclusions according to the comprehensive system evaluation results. The results with high heterogeneity were analyzed by segmental exclusion method to explore the possible sources of heterogeneity and analyze the sensitivity of the results accordingly. If the number of randomized controlled trials for an indicator was more than five, the bias on publication should be assessed and a funnel plot should be used. All analysis methods were based on PRISMA.

## Results

### Data overview

**Fig. 1:**
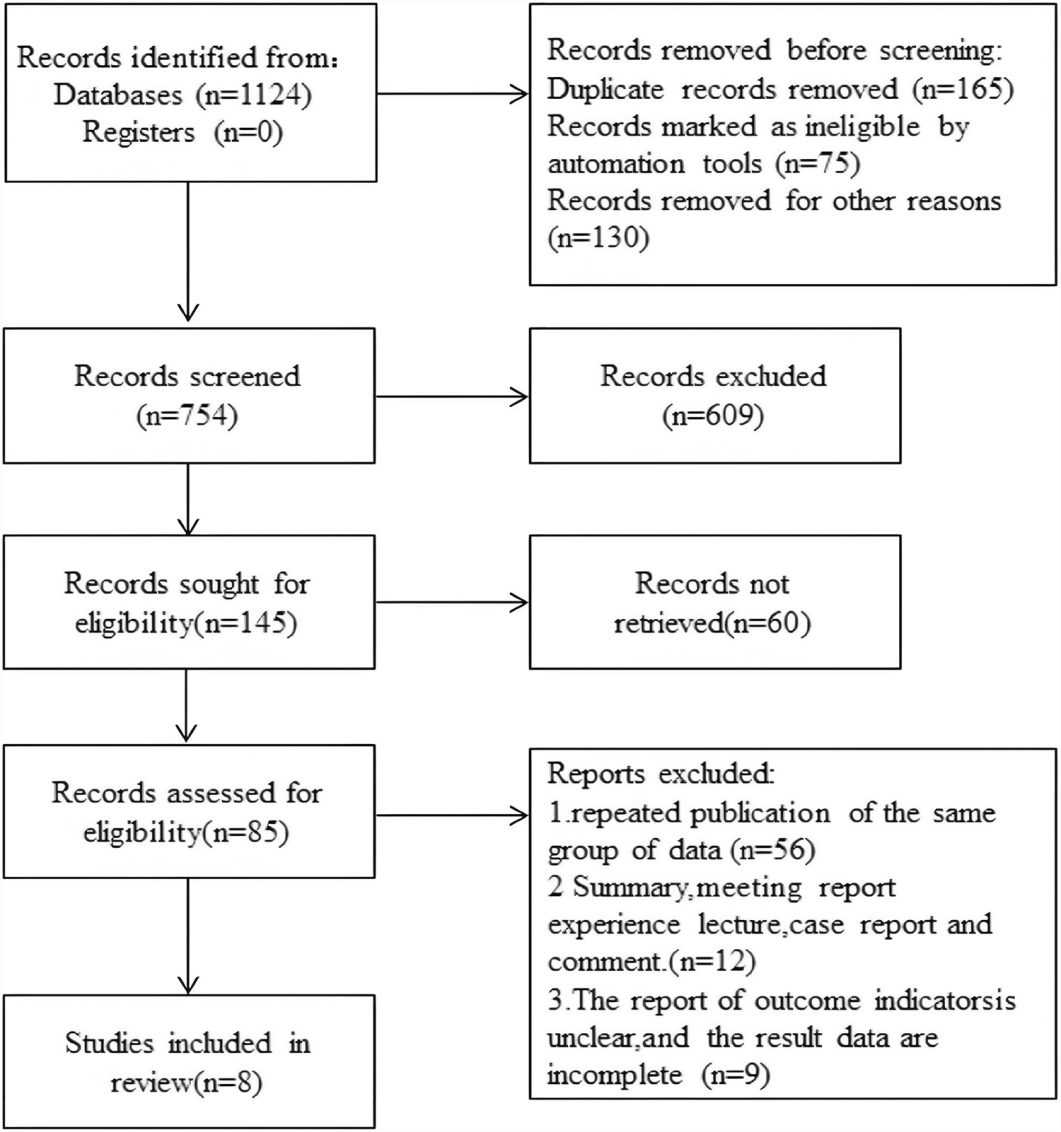
Literature sift process

In this study, 1124 related documents were initially retrieved, including 542 from PubMed, 249 from Embase, 152 from Ovid-Medline, and 181 from other databases. 370 articles were initially excluded based on the criteria; after reading titles and abstracts, 609 papers were excluded. After the rest of the papers were read in full, another 60 papers were deleted. After reading the full text of the literature in a careful way, 75 papers were excluded, and 8 researches which was matched the inclusion criteria were included eventually. The literature sift process is shown in Fig. 1.

### Basic features of the included researches

All of 981 sufferers were included in the meta-analysis, including 612 cases in the case group and 36 cases in the control one. The main features of the included researches are listed in Table 1.

**Table 1:** Basic features of the included researches

Included studies	Type of study	Number of cases		Age		Gender: (male/female)	
		Case group	Control group	Case group	Control group	Case group	Control group
Chuang2019 [10]	Case–control study	75	75	42.6 ± 3.7	43.8 ± 3.0	65/10	62/13
Nowicki2020 [11]	Case–control study	86	86	Not described	Not described	Not described	Not described
Zhang2013 [12]	Case–control study	75	23	32.11 ± 5.80	33.50 ± 24.71	Not described	Not described
Han2013 [13]	Case–control study	56	25	Not described	Not described	Not described	Not described
Chen2013 [14]	Case–control study	199	75	54.5	Not described	172/27	Not described
Luo2009 [15]	Case–control study	45	15	46.80 ± 9.50	48.40 ± 10.20	31/14	10/5
Yu2008 [16]	Case–control study	46	40	52.50 ± 10.30	54.50 ± 9.60	41/5	25/5
Li2006 [17]	Case–control study	30	30	51.00 ± 8.00	51.00 ± 9.00	22/8	20/10

### Quality assessment of included researches

The each article quality was assessed using the Newcastle–Ottawa Scale scoring. The results showed that all eight articles had a low risk of bias and matched the demands of follow-up analysis. See Table 2.

**Table 2:** Quality assessment of included researches

Included studies	1	2	3	4	5	6	7	8	9	Total scores
Chuang2019 [10]	Yes	No	Yes	Yes	Yes	No	Yes	No	Yes	7
Nowicki2020 [11]	Yes	Yes	Yes	Yes	Yes	No	Yes	Yes	Yes	8
Zhang2013 [12]	Yes	Yes	No	Yes	Yes	No	Yes	Yes	Yes	7
Han2013 [13]	Yes	Yes	Yes	Yes	Yes	No	Yes	Yes	Yes	8
Chen2013 [14]	Yes	No	No	Yes	Yes	No	Yes	Yes	Yes	6
Luo2009 [15]	Yes	Yes	Yes	Yes	Yes	No	Yes	Yes	Yes	8
Yu2008 [16]	Yes	Yes	Yes	Yes	Yes	No	Yes	Yes	Yes	8
Li2006 [17]	Yes	Yes	Yes	Yes	Yes	No	Yes	Yes	Yes	8

### *Meta*-analysis

#### Obstructive sleep apnea syndrome and serum cystatin C

**Fig. 2:**
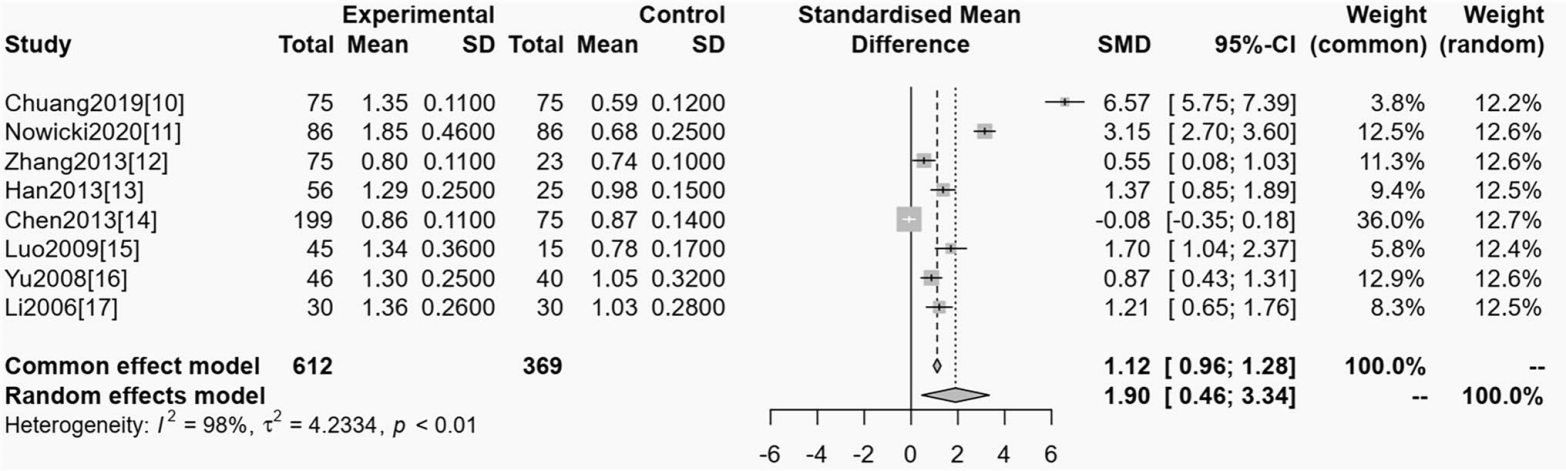
Forest plot of meta-analysis of the relationship between OSAS and serum cystatin C

Among the included studies, 8 trials reported the relationship between OSAS and serum cystatin C. The homogeneity test showed (*P* < 0.01, *I*^*2*^ = 98%), showing that it had heterogeneity, so random impacts model was used for statistical analyzing. From the meta-analysis result, it could be known that the serum cystatin C levels of OSAS was obviously greater than the control one, and the distinction had statistical significance (OR = 1.12, 95% CI 0.96–1.28, *P* < 0.01). See Fig. 2.

#### OSAS and serum creatinine

**Fig. 3:**
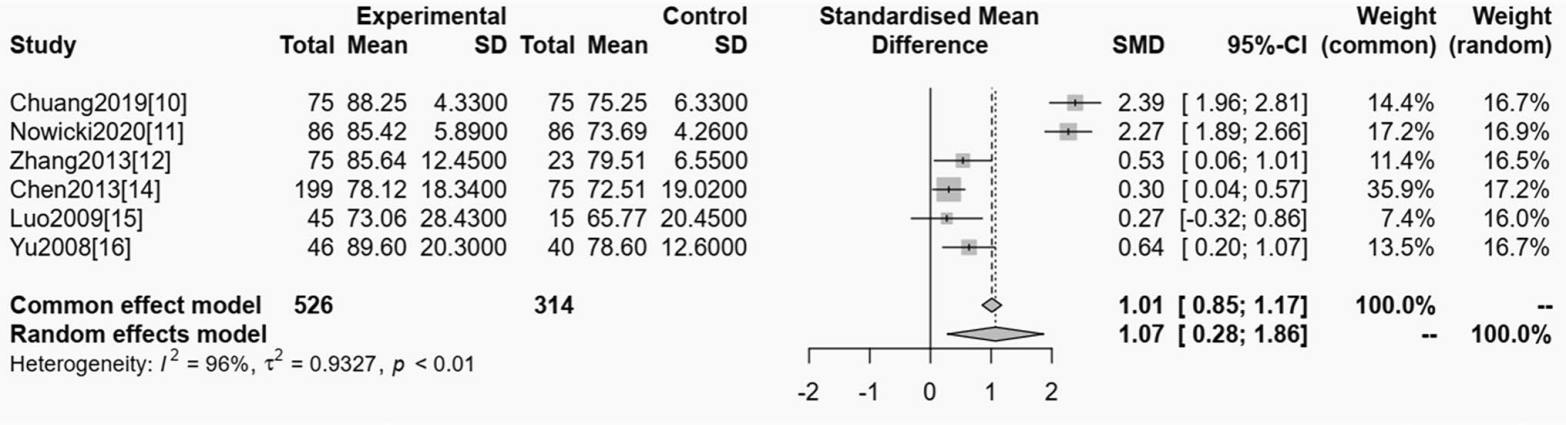
The forest plot of meta-analysis of the relationship between OSAS and serum creatinine

Among the included studies, six trials reported the relationship between OSAS and serum creatinine, and homogeneity test showed (*P* < 0.01, *I*^*2*^ = 96%), showing that it had heterogeneity, so random impacts were used model for statistical analyzing. From the meta-analysis result, it could be known that the serum creatinine level of OSAS was obviously greater than the control one, and the distinction had statistical significance (OR = 1.01, 95% CI 0.85 ~ 1.17, *P* < 0.01). See Fig. 3.

#### OSAS and serum urea nitrogen

**Fig. 4:**
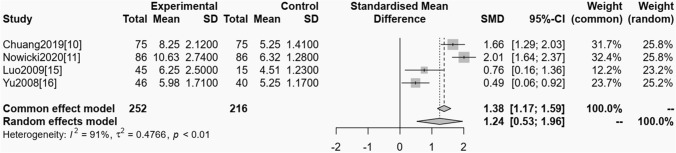
The meta-analysis forest plot of the relationship between OSAS and serum urea nitrogen

Among the included studies, 4 trials reported the relationship between OSAS and serum urea nitrogen. The homogeneity test showed (*P* < 0.01, *I*^*2*^ = 91%), showing that it had heterogeneity, so random impacts model was used model for statistical analyzing. From the meta-analysis result, it could be showed that serum urea nitrogen of OSAS was obviously greater than the control one, and the distinction had statistical significance (OR = 1.38, 95% CI 01.17 ~ 1.59, *P* < 0.01). See Fig. 4.

#### Sensitivity analysis

**Fig. 5:**
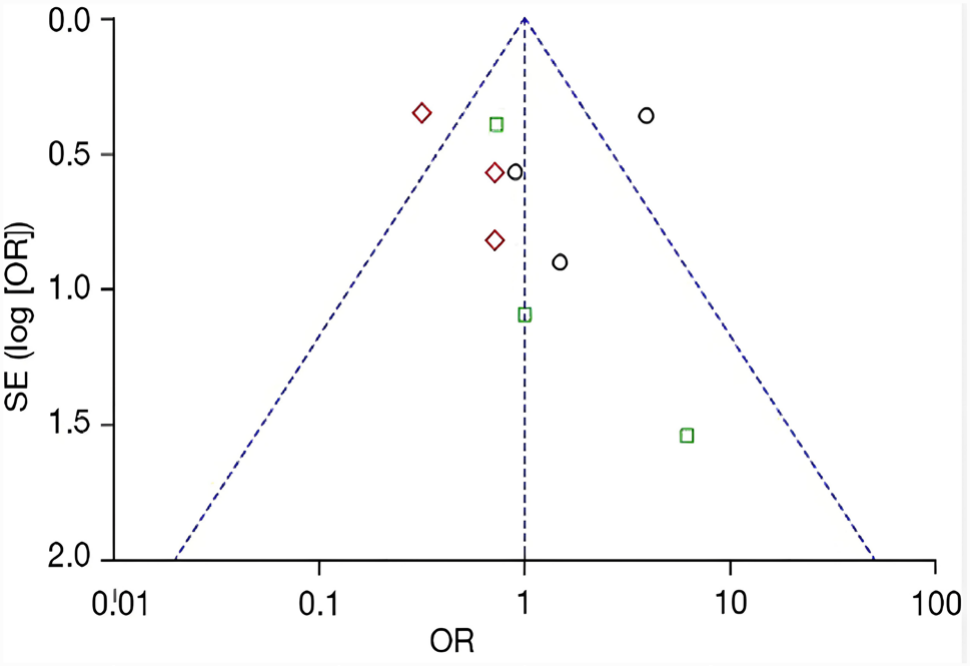
Funnel plot of sensitivity analysis

Evidence of publication bias were not detected using the Begg’s test and Egger’s test, and the results of the funnel plot showed left–right symmetry, so it had no bias on publication in this research. See Fig. 5.

## Discussion

Obstructive sleep apnea syndrome (OSAS) is a sleep-breathing disorder of unknown etiology, characterized by nocturnal snoring with apnea and daytime sleepiness. Repeated episodes of nocturnal hypoxia and hypercapnia caused by apnea can lead to hypertension, coronary heart disease, diabetes mellitus, cerebrovascular disease and other complications, as well as traffic accidents, and even sudden death at night. Thus, obstructive sleep apnea syndrome is a potentially lethal sleep breathing disorder [10]. Clinical data show that the incidence of sleep apnea syndrome in adults is 15–24%. Sleep apnea syndrome in adults is also an important risk factor for many diseases such as kidney injury, cardiovascular disease, cerebrovascular disease, asthma and pulmonary edema [18]. Many theories explain the reasons for the development of multiple disorders, among which oxidative stress, hypoxemia, inflammatory response and hypertension due to adult sleep apnea syndrome are the more recognized causes. In addition, obstructive sleep apnea syndrome can cause chronic hypoxia, which is more severe when sleep apnea occurs, and not only increases the risk of cardiovascular disease, but also leads to chronic renal hypoxia, which leads to a range of hypoxia-related pathologic damage [19]. The renal tissue that is most sensitive to hypoxia is the renal tubules, so patients who fight apnea tend to experience symptoms of interstitial damage such as increased nocturia, followed by gradual onset of renal hypoplasia over time. Sleep apnea syndrome and chronic kidney disease have a bidirectional relationship, sleep apnea syndrome promotes chronic kidney disease to develop into uremia, and 50–70% of end-stage renal disease is accompanied by sleep apnea syndrome [20, 21]. It indicates that there is a close relationship between the two diseases.

It was verified that there was no publication bias in the studies enrolled in this study, and the sensitivity analysis also showed that the results of this study were more stable. Therefore, the results of this study can be considered more reliable. Among all eight studies included in this study, the results showed that the serum cystatin C (OR = 1.12, 95% CI 0.96 ~ 1.28, *P* < 0.01), creatinine (OR = 1.12, 95% CI 0.96 ~ 1.28, *P* < 0.01), and urea nitrogen (OR = 1.38, 95% CI 01.17 ~ 1.59) levels of obstructive sleep apnea syndrome were significantly higher than those of control group. Levels were significantly higher than those in the control group. Serum cystatin C can freely pass through the glomerular filtration membrane, is almost completely reabsorbed in the proximal tubule and rapidly metabolized and decomposed, and will not re-enter the blood circulation; therefore, the kidney is the only organ that removes cystatin C from the blood circulation, and its blood concentration is completely dependent on the glomerular filtration rate, which has the best correlation with the glomerular filtration rate, and it has a good early assessment and diagnostic value of renal pathology [22, 23]. Creatinine is a small molecule that is filtered through the glomerulus and is rarely reabsorbed in the renal tubules, and almost all of the creatinine produced in the body each day is excreted in the urine, generally independent of urine output. Pathological increase in blood creatinine represents a decrease in glomerular filtration rate and renal detoxification function, suggesting that renal function has been impaired, and as the creatinine index continues to rise, the patient’s renal function will become more and more severely impaired [24]. Urea nitrogen is a nitrogenous compound in plasma other than protein, which is excreted from the body by glomerular filtration. In renal insufficiency loss of compensation, serum urea nitrogen will be elevated. Therefore, it is used clinically as an indicator of glomerular filtration function. When renal function is mildly impaired, urea nitrogen can be unchanged; when urea nitrogen is elevated, it indicates that 60 ~ 70% of the kidney has been damaged, so blood urea nitrogen can not be used as an early functional indicator of renal disease, but it has a special value for the diagnosis of renal insufficiency, especially uremia, and the degree of its increase is proportional to the severity of the disease, so it has an important significance in the determination of the disease and the estimation of the prognosis [25, 26].

A conclusion based on a result of a unitary study is hard to show the nature of things, and the result may be accidental due to the impacts of multiple factors. Meta-analysis is a study method for systematic analysis and quantitative synthesis of multiple independent study results with the same study goal. The aim of meta-analysis is the improvement of the efficiency of statistical testing, the evaluation of the inconsistencies or contradictions of the study results, and to find the deficiencies of a unitary research by processing a large number of literatures, and it is not a limitation with the amount of researches. And it has a significant function with diagnosis, therapy, assessment of risk, the intervention of prevention, health services, and decision-making in clinical [27, 28]. Because of the small sample size of clinical instance researches on OSAS and kidney injury, this article has provided a quantitative mean impact or correlation combined with multiple researches, making the results of every research more quantitative and comprehensive, and the conclusions more dependable and comprehensive. OSAS promotes the occurrence of renal injury through multiple mechanisms; however, the specific mechanism has not been fully elucidated. The apnea, chronic intermittent hypoxia and sleep structure disorder caused by obstructive sleep apnea syndrome can lead to a lot of pathophysiological changes such as hemodynamic changes, increased sympathetic nerve activity, and endothelial dysfunction, all of which may be the basis of the pathogenesis of renal injury [29]. Therefore, obstructive sleep apnea syndrome can affect and aggravate renal function damage in patients through various ways. In clinical practice, timely identification and giving intervention measures are of great significance to reduce the mortality ratio and improve the prognosis of sufferers.

## Conclusion

In summary, this study has found that obstructive sleep apnea syndrome is closely related to kidney injury, and the two may be risk factors for each other. The strength of the research is the analysis of the occurrence of renal injury in OSAS, and the therapy in the future provided for a solid foundation. Nevertheless, it had also disadvantages in the research: (1) in addition to obstructive sleep apnea syndrome as the mainly observational factors in the included research, it had many other risk factors for renal injury, and it is hard for using meta-analysis to correct or exclude the impact of other therapy factors; (2) the meta-analysis is not an experimental research, so there may be multiple biases; (3) all of the included researches are hospital-based instance-control researches. The quality of all studies could not be assessed according to the NOS scale.

## Data Availability

No datasets were generated or analysed during the current study.
